# Combined toxicities of cadmium and five agrochemicals to the larval zebrafish (*Danio rerio*)

**DOI:** 10.1038/s41598-022-20364-8

**Published:** 2022-09-26

**Authors:** Guixian Hu, Hao Wang, Yujie Wan, Liangliang Zhou, Qiang Wang, Minghua Wang

**Affiliations:** 1 Department of Pesticide Science, College of Plant Protection, State & Local Joint Engineering Research Center of Green Pesticide Invention and Application, Nanjing Agricultural University, Nanjing, 210095 China; 2 State Key Laboratory for Managing Biotic and Chemical Threats to the Quality and Safety of Agro-Products, Institute of Agro-Product Safety and Nutrition, Zhejiang Academy of Agricultural Sciences, Hangzhou, 310021 China

**Keywords:** Environmental sciences, Environmental impact

## Abstract

Different pollutants usually co-exist in the natural environment, and the ecological and health risk assessment of agrochemicals needs to be carried out based on the combined toxicological effects of pollutants. To examine the combined toxicity to aquatic organisms, the effects of cadmium (Cd) and five pesticides (acetamiprid, carbendazim, azoxystrobin, chlorpyrifos, and bifenthrin) mixture on zebrafish (*Danio rerio*) larvae were assessed. The data from the 96-h toxicity test indicated that bifenthrin possessed the highest toxicity to *D. rerio* with the LC_50_ value of 0.15 mg L^−1^, followed by chlorpyrifos (0.36 mg L^−1^) and azoxystrobin (0.63 mg L^−1^). Cd (6.84 mg L^−1^) and carbendazim (8.53 mg L^−1^) induced the intermediate toxic responses, while acetamiprid (58.39 mg L^−1^) presented the lowest toxicity to the organisms. Pesticide mixtures containing chlorpyrifos and bifenthrin or acetamiprid and carbendazim showed synergistic impacts on the zebrafish. Besides, two binary combinations of Cd-acetamiprid and Cd-chlorpyrifos also displayed a synergistic effect on *D. rerio*. Our results offered a better idea of the mixed ecological risk assessment of Cd and different agricultural chemicals to aquatic organisms. Our findings better interpreted how the interaction between Cd and various agrochemicals changed their toxicity to aquatic vertebrates and provided valuable insights into critical impacts on the ecological hazard of their combinations.

## Introduction

The water ecosystem is often simultaneously threatened by numerous environmental pollutants^1^. Heavy metals in freshwater environments are of great concern because they are bio-accumulative, non-biodegradable, and toxic to aquatic biota^2,3^. Cadmium (Cd) is extensively found in water bodies, and its presence is significantly correlated with a series of human activities, such as mining, waste emission, fertilizer application, etc^4^. Besides, Cd and pesticides often co-exist in the same water samples, especially in areas with intensive agricultural and industrial activities^5^. Therefore, aquatic organisms are usually challenged by many contaminants, and exposure to multi-component mixtures may induce substantial toxic impacts, even if the concentrations of these single compounds are lower than their no-observed-effect concentration (NOEC)^6^. Currently, the number of studies addressing the mixture toxicity of organic pollutants (agrochemicals) or inorganic pollutants (heavy metals) is increasing^7,8^. Nevertheless, studies on mixture exposure of heavy metals and agrochemicals are still scarce^9^. Consequently, it is essential to better comprehend the interactive impacts of organic–inorganic pollutant mixtures on non-target organisms.

Pollutants affect aquatic organisms in the water environment, and fish are essential bio-indicators^10^. As a well-established model organism, zebrafish (*Danio rerio*) are often used in toxicological studies because of several beneficial features, such as small size, rapid reproductive capacity, and transparency in early life stages^11,12^. Besides, it is possible to explore the embryonic development of *D. rerio* through morphological observations of various endpoints when exposed to the chemical during embryogenesis^13^. The early life stages of zebrafish are especially vulnerable to environmental contaminations^14^. Exposure to low levels of chemicals during the embryonic stage of zebrafish can affect their later developmental stages^15^. Hence, the toxicity assessments derived from the early life stage of *D. rerio* can highly reflect the potential biological impacts of toxicants.

Risk assessment usually focuses on the toxicity of individual chemicals to the zebrafish. However, environmental pollutants usually co-exist as complex combinations in the natural environment^16^. Therefore, it is more accurate to assess the mixture toxicity of pollutants^17,18^. Owing to the wide use of acetamiprid, carbendazim, azoxystrobin, chlorpyrifos, and bifenthrin, their possible effects on aquatic organisms are of great attention^19^. Moreover, Cd and the five pesticides are often found in the same water bodies^20,48^. Consequently, more investigations are required to better explore their potential ability to produce additive, synergistic, or antagonistic toxicities in the environment^18,21^. In our current study, we evaluated the combined toxic effects of Cd and different agrochemicals on *D. rerio*, and the result could help better comprehend the possible environmental risk of co-occurrence of heavy metals and agrochemicals in the water ecosystem.

## Materials and methods

### Ethical statement

For the protection of animal welfare, all the animal maintenance and procedures were in accordance with recommendations established by the Animal Ethics Committee of Nanjing Agricultural University, as well as the Laboratory Animal Guideline for Ethical Review of Animal Welfare from China (GB/T 35892-2018). All procedures were carried out under deep anesthesia, and all efforts were made to minimize suffering. The study was reported in accordance with ARRIVE guidelines (https://arriveguidelines.org).

### Fish breeding and embryo production

Adult zebrafish (AB strain and about 6 months old) were purchased from China Zebrafish Resource Center (Hubei, China). Fish were maintained at approximately 27 ± 1 °C in a flow-through system under a light–dark cycle of 14/10 h. The zebrafish were fed two times a day with a commercial diet (Tetra, Germany) and freshly hatched brine shrimps (*Artemia nauplii*). The test equipment and aquariums were cleaned every week.

Female and male adult fish (female/male ratio was 1/2) were separated by isolation boards in spawning boxes overnight. In the following morning, spawning was triggered once the light was turned on, and the isolation boards were removed. When the spawning finished, and the eggs were collected and observed under a microscope after 30 min.

Fertilized embryos were washed and placed into a crystallizing dish (bottom diameter of 15 cm) containing reconstituted water^49^. The external conditions, including temperature and light cycle, of the embryos were maintained the same as the breeding environment of adults. In addition, larvae of 5 dpf (days post-fertilization) were used in the toxicity test.

### Chemicals and reagents

One heavy metal Cd and five pesticides, including acetamiprid, azoxystrobin, bifenthrin, carbendazim, and chlorpyrifos, were evaluated in this study (Table 1). To prepare the Cd stock solution of 100,000 mg L^−1^, CdCl_2_·2.5H_2_O was dissolved in deionized water in sealed polyethylene containers. Stock solutions of each pesticide were prepared using N,N-dimethylformamide (DMF) and 10% Tween-80 and preserved at 4 °C for up to 30 days. Before specified assays, suitable working solutions were further prepared using the stock solutions and reconstituted water.

**Table 1:** Detailed information about the chemical tested in this study.

Chemicals	Category	Purity (%)	Mode of action	Aqueous photolysis DT_50_ (days) at pH 7	Manufacturer
Cadmium (CdCl_2_·2.5H_2_O)	Heavy metal	≥ 99	Calcium channel blocker	10–30 years in organisms	Jinshanting New Chemical Industrial Group (Shanghai, China)
Acetamiprid	Neonicotinoid insecticide	99	Nicotinic acetylcholine receptors agonist	34	Jiangsu Repont Agrochemical Co., Ltd. (Changzhou, China)
Azoxystrobin	Strobilurin fungicide	97	Respiratory inhibition of fungal mitochondria	8.7	Jiangsu Frey Agrochemicals Co., Ltd. (Zhenjiang, China)
Bifenthrin	Pyrethroid insecticide	98	Activates voltage gated sodium channels	12	Nanjing Red Sun Chemical Co., Ltd. (Nanjing, China)
Carbendazim	Benzimidazole fungicide	98	C14α-demethylase inhibitor	350	Jiangsu Jialong Chemical Co., Ltd. (Lianyungang, China)
Chlorpyrifos	Organophosphate insecticide	98	Inhibits acethylcholinesterase	29.6	Shandong Huayang Agrochemical Group Co., Ltd. (Ningyang, China)

### Toxicity assays of single compounds

The acute toxicity assay of larval fish was conducted according to our previous protocol^22^. The healthy larval fish of 5 dpf were randomly chosen and put into 24-well plates, and there were one larva and 2 mL of exposure solution in each well. Both blank control and solvent control were established. The same concentrations of DMF and Tween-80 as those of the highest dosage solution for each pesticide were used in the solvent control. Each test concentration or control was tested in three replicates using a 24-well plate as a replicate. Four to six doses with a geometrical ratio that produced a death rate of 10–90% were evaluated for each compound. All 24-well plates covered by transparent lids were incubated at 27 ± 1 °C with a 14/10-h ligh/dark photoperiod for 96 h. The exposure solution was refreshed every 12 h to maintain the approximate concentration of chemical and water quality. No feed was provided to larval fish during the toxicity evaluations. Fish without a heartbeat was considered as dead. Mortality of every exposure concentration was registered after exposure for 96 h.

### Joint toxicity assays

The joint toxic effects were assessed using *D. rerio* larvae of 5 dpf, which were conducted at equivalent concentration and equitoxic ratio measurements, respectively. The toxic impacts of the single chemicals were directly compared with their mixtures. For every treated concentration, the test was conducted in triplicate.

#### Equivalent concentration measurement

For the equivalent concentration mixture exposures, the initial dose (1 ×) of each tested chemical was 5% of the 96-h LC_50_ of the higher (or the highest) toxicity chemical when existing individually. The doses of every single chemical in the mixture were then sequentially doubled (1 ×, 2 ×, 4 ×, 8 ×, 16 ×, 32 ×) to yield the six concentrations tested. The dose of binary, ternary, quaternary, and quinquenary mixtures remained unchanged (1:1, 1:1:1, 1:1:1:1, or 1:1:1:1:1), while the total concentration of the mixture was systematically changed.

#### Equitoxic ratio measurement

Fish were exposed to serial dilutions of each chemical at a fixed equitoxic constant mixture ratio (the same lethal toxicity for each compound to larval fish) of 1:1 (50% of the 96 h-LC_50_ of each pesticide), 1:1:1 (33.3% of the 96 h-LC_50_ of each pesticide), 1:1:1:1 (25% of the 96 h-LC_50_ of each pesticide), 1:1:1:1:1 (20% of the 96 h-LC_50_ of each pesticide), and 1:1:1:1:1:1 (16.7% of the 96 h-LC_50_ of each pesticide), respectively, based on the detected LC_50_ values of single compounds. Four to six dilutions with a geometrical ratio of each compound with their two-, three-, four-, five-, and six-component mixtures were examined.

### Statistical analysis

The acute toxicities of chemicals to *D. rerio* were evaluated by a probit analysis using a previously developed program^23^. Toxicity was deemed significantly different if the 95% fiducial limits of two LC_50_ values did not overlap (*P* < 0.05). The interplay patterns of chemicals were identified using an additive index (AI) according to the LC_50_ data acquired from both single compounds and their combinations^24^.

$$ S = \, \left( {A{\text{m}}/A{\text{i}}} \right) \, + \, \left( {B{\text{m}}/B{\text{i}}} \right) $$

where *S* refers to the sum of biological activities of *A* and *B*; *A*m is the LC_50_ for chemical *A* in the mixture, *A*i refers to the LC_50_ for chemical *A* when used alone; *B*m represents the LC_50_ for chemical *B* in the mixture, and *B*i is the LC_50_ for chemical *B* when used alone.

- *S* values were then used to calculate the AI.

The AI value was calculated from the sum of *S* using the equations as follows:

- If *S* < 1.0, then the AI = 1/*S* − 1.
- If *S* ≥ 1.0, then the AI = 1 − *S*.

Mixture toxicity was classified into antagonistic effect (AI ≤ − 0.2), additive effect (− 0.2 < AI ≤ 0.25), or synergetic effect (AI > 0.25). A higher AI value indicates a higher pesticide synergism^25^.

### Ethics approval and consent to participate

The authors confirm that the national laws regarding animal protection were followed.

## Results

The doses of chemicals used in our current work would be expected to be almost improbable in the environment, which may only be observed when specific events happen, such as direct application or unfortunate leakage. Nevertheless, we cannot exclude that the piscine population and occupationally exposed workers could be exposed accidentally to these toxins within this range of concentrations.

### Acute toxicities of single agrochemicals

The toxicities of different compounds to *D. rerio* were remarkably different. The LC_50_ values of the six tested chemicals to the organisms ranged from 0.15 to 58.39 mg L^−1^. The greatest toxicity to *D. rerio* was detected from bifenthrin with an LC_50_ value 0.15 mg L^−1^, followed by chlorpyrifos and azoxystrobin with LC_50_ values of 0.36 and 0.63 mg L^−1^, respectively. Cd and carbendazim induced intermediate toxic responses, and their LC_50_ values were 6.84 and 8.53 mg L^−1^, respectively. However, acetamiprid exhibited the lowest toxicity to the organisms, with an LC_50_ value of 58.39 mg L^−1^. Based on the LC_50_ values, the toxicity of bifenthrin was 389.3 times higher compared with acetamiprid. The toxicities of these compounds to *D. rerio* were ranked as follows: bifenthrin > chlorpyrifos > azoxystrobin > Cd, carbendazim > acetamiprid.

### Toxic impacts of chemical mixtures

#### Equivalent concentration assay

##### Toxic effects of two-component mixtures

The LC_50_ values of different two-component mixtures after 96 h of exposure were calculated to explore the combined toxic impacts of Cd and five agrochemicals on *D. rerio*. The mixtures of Cd-acetamiprid and Cd*-*chlorpyrifos produced a synergistic effect, and their AIs ranged from 0.51 to 1.14. Nevertheless, the AIs of mixtures of Cd-carbendazim, Cd-azoxystrobin, and Cd-bifenthrin ranged from − 0.63 to − 0.29 after 96 h of exposure, indicating antagonistic effects (Table 2).

**Table 2:** Mixture toxicity of Cd and five pesticides with equivalent concentration to the larvae of *D. rerio*.

Chemical combinations	LC_50_ (95%FL) mg L^−1^	AI value	Mixture effect
**Binary mixtures**			
Cd-ACT	4.08 (2.43–5.68)	0.51	Synergism
Cd-CAR	5.37 (4.09–6.89)	− 0.42	Antagonism
Cd-AZO	0.94 (0.68–1.25)	− 0.63	Antagonism
Cd-CHL	0.16 (0.073–0.28)	1.14	Synergism
Cd-BIF	0.19 (0.10–0.32)	− 0.29	Antagonism
**Ternary mixtures**			
Cd-ACT-CAR	0.94 (0.65–1.38)	2.79	Synergism
Cd-ACT-AZO	1.03 (0.78–1.29)	− 0.80	Antagonism
Cd-ACT-CHL	0.25 (0.11–0.42)	0.36	Synergism
Cd-ACT-BIF	0.13 (0.081–0.21)	0.13	Additive
Cd-CAR-AZO	0.85 (0.59–1.16)	− 0.57	Antagonism
Cd-CAR-CHL	0.23 (0.11–0.37)	0.43	Synergism
Cd-CAR-BIF	0.35 (0.19–0.58)	− 1.43	Antagonism
Cd-AZO-CHL	0.39 (0.24–0.61)	− 0.76	Antagonism
Cd-AZO-BIF	0.18 (0.096–0.29)	− 0.52	Antagonism
Cd-CHL-BIF	0.037 (0.015–0.063)	1.82	Synergism
**Quaternary mixtures**			
Cd-ACT-CAR-AZO	0.26 (0.17–0.38)	1.06	Synergism
Cd-ACT-CAR-CHL	0.21 (0.098–0.35)	0.56	Synergism
Cd-ACT-CAR-BIF	0.10 (0.054–0.17)	0.43	Synergism
Cd-ACT-AZO-CHL	0.19 (0.10–0.31)	0.16	Additive
Cd-ACT-AZO-BIF	0.23 (0.12–0.37)	− 0.94	Antagonism
Cd-ACT-CHL-BIF	0.031 (0.016–0.048)	2.36	Synergism
Cd-CAR-AZO-CHL	0.47 (0.21–0.75)	− 1.18	Antagonism
Cd-CAR-AZO-BIF	0.21 (0.10–0.35)	− 0.79	Antagonism
Cd-CAR-CHL-BIF	0.046 (0.029–0.068)	1.24	Synergism
Cd-AZO-CHL-BIF	0.031 (0.014–0.048)	1.89	Synergism
**Quinquenary mixtures**			
Cd-ACT-CAR-AZO-CHL	0.13 (0.058–0.22)	0.65	Synergism
Cd-ACT-CAR-AZO-BIF	0.061 (0.039–0.086)	0.92	Synergism
Cd-ACT-CAR-CHL-BIF	0.026 (0.011–0.045)	2.96	Synergism
Cd-ACT-AZO-CHL-BIF	0.031 (0.014–0.048)	1.88	Synergism
Cd-CAR-AZO-CHL-BIF	0.036 (0.021–0.054)	1.46	Synergism
**Senary mixture**			
Cd-ACT-CAR-AZO-CHL-BIF	0.021 (0.010–0.36)	3.21	Synergism

*FL* fiducial limit, *AI* additive index, *ACT* acetamiprid, *CAR* carbendazim, *AZO* azoxystrobin, *CHL* chlorpyrifos, *BIF* bifenthrin.

##### Toxic impacts of three-component mixtures

Four three-component mixtures of Cd-acetamiprid-carbendazim, Cd-acetamiprid-chlorpyrifos, Cd-carbendazim-chlorpyrifos, and Cd-chlorpyrifos-bifenthrin displayed synergistic responses, and their AIs ranged from 0.36 to 2.79 after 96 h of exposure. The calculated AIs for the five three-component mixtures of Cd-acetamiprid-azoxystrobin, Cd-carbendazim-azoxystrobin, Cd-carbendazim-bifenthrin, Cd-azoxystrobin-chlorpyrifos, and Cd-azoxystrobin-bifenthrin ranged from − 1.43 to − 0.52 after 96 h of exposure, showing antagonistic reactions. In contrast, the three-component mixture of Cd-acetamiprid-bifenthrin showed an additive reaction, with an AI of 0.13 after 96 h of exposure (Table 2).

##### Toxic impacts of four-component mixtures

For the six four-component mixtures of Cd-acetamiprid-carbendazim-azoxystrobin, Cd-acetamiprid-carbendazim-chlorpyrifos, Cd-acetamiprid-carbendazim-bifenthrin, Cd-acetamiprid-chlorpyrifos-bifenthrin, Cd-carbendazim-chlorpyrifos-bifenthrin, and Cd-azoxystrobin-chlorpyrifos-bifenthrin, their AIs ranged from 0.43 to 2.36 after 96 h of exposure, exhibiting synergistic reactions. In contrast, the calculated AIs for three four-component mixtures of Cd-acetamiprid-azoxystrobin-bifenthrin, Cd-carbendazim-azoxystrobin-chlorpyrifos, and Cd-carbendazim-azoxystrobin-bifenthrin ranged from − 1.18 to − 0.79 after 96 h of exposure, showing antagonistic reactions. The four-component mixture of Cd-acetamiprid-azoxystrobin-chlorpyrifos showed an additive reaction, and its AI was 0.16 after 96 h of exposure (Table 2).

##### Toxic impacts of five- and six-component mixtures

For all the five-component mixtures of Cd-acetamiprid-carbendazim-azoxystrobin-chlorpyrifos, Cd-acetamiprid-carbendazim-azoxystrobin-bifenthrin, Cd-acetamiprid-carbendazim-chlorpyrifos-bifenthrin, Cd-acetamiprid-azoxystrobin-chlorpyrifos-bifenthrin, and Cd-carbendazim-azoxystrobin-chlorpyrifos-bifenthrin, their AIs ranged from 0.65 to 2.96 after 96 h of exposure, exhibiting synergistic reactions. Besides, the calculated AI for the six-component mixture of Cd-acetamiprid-carbendazim-azoxystrobin-chlorpyrifos-bifenthrin was 3.21 after 96 h of exposure, also suggesting a synergistic response (Table 2).

#### Equitoxic ratio assay

##### Toxic impacts of two-component mixtures

Two two-component mixtures of Cd-acetamiprid and Cd-chlorpyrifos still showed synergistic reactions, and their AIs ranged from 0.69 to 1.92 after 96 h of exposure. Meanwhile, the two two-component mixtures of Cd-carbendazim and Cd-azoxystrobin still showed an antagonistic reaction, and their AIs ranged from − 0.87 to − 0.40 after 96 h of exposure. The calculated AI for the two-component mixture of Cd-bifenthrin was − 0.026 after 96 h of exposure, suggesting an additive response (Table 3).

**Table 3:** Mixture toxicity of Cd and five pesticides with equitoxic ratio to the larvae of *D. rerio*.

LC_50_ (95%FL) mg L^−1^						AI value	Mixture effect
Cd	ACT	CAR	AZO	CHL	BIF		
**Binary mixtures**							
2.03 (1.57–2.69)	17.33 (13.40–22.97)					0.69	Synergism
4.76 (3.69–5.96)		5.94 (4.60–7.41)				− 0.4	Antagonism
6.38 (5.14–7.81)			0.59 (0.47–0.72)			− 0.87	Antagonism
1.17 (0.74–1.69)				0.062 (0.038–0.089)		1.92	Synergism
3.51 (2.39–3.86)					0.076 (0.052–0.085)	− 0.026	Additive
**Ternary mixtures**							
0.53 (0.41–0.69)	4.52 (3.50–5.89)	0.66 (0.51–0.86)				3.30	Synergism
4.89 (3.65–6.47)	41.75 (31.16–55.24)		0.45 (0.34–0.59)			− 1.15	Antagonism
1.26 (0.82–1.74)	10.76 (7.00–14.86)			0.066 (0.043–0.092)		0.81	Synergism
1.93 (1.34–2.69)	16.48 (11.44–22.96)				0.042 (0.026–0.059)	0.18	Additive
3.38 (2.71–4.25)		4.21 (3.38–5.30)	0.31 (0.24–0.39)			− 0.48	Antagonism
1.39 (0.64–2.47)		1.73 (0.79–3.08)		0.073 (0.034–0.13)		0.64	Synergism
6.61 (5.09–8.71)		8.24 (6.34–10.86)			0.14 (0.11–0.19)	− 1.89	Antagonism
3.47 (1.94–5.36)			0.32 (0.19–0.49)	0.18 (0.10–0.28)		− 0.52	Antagonism
3.04 (1.73–3.67)			0.28 (0.15–0.34)		0.067 (0.037–0.080)	− 0.33	Antagonism
0.62 (0.28–1.09)				0.033 (0.014–0.057)	0.014 (0.0061–0.024)	2.68	Synergism
**Quaternary mixtures**							
0.54 (0.38–0.89)	4.61 (3.24–7.59)	0.67 (0.47–1.11)	0.049 (0.035–0.082)			2.16	Synergism
1.17 (0.74–1.69)	9.98 (6.31–14.43)	1.46 (0.92–2.11)		0.062 (0.038–0.089)		0.46	Synergism
1.04 (0.68–1.52)	8.88 (5.81–12.97)	1.29 (0.85–1.90)			0.023 (0.014–0.033)	0.65	Synergism
1.21 (0.71–1.86)	10.33 (6.06–15.88)		0.11 (0.065–0.17)	0.064 (0.037–0.097)		0.41	Synergism
2.71 (1.84–3.92)	23.14 (15.70–33.46)		0.25 (0.16–0.36)		0.059 (0.040–0.086)	− 0.58	Antagonism
0.43 (0.23–0.68)	3.67 (1.96–5.81)			0.023 (0.012–0.036)	0.0094 (0.0050–0.015)	2.97	Synergism
2.51 (1.67–3.58)		3.13 (2.08–4.46)	0.23 (0.15–0.33)	0.13 (0.087–0.19)		− 0.47	Antagonism
2.29 (1.76–2.81)		2.85 (2.19–3.50)	0.21 (0.16–0.25)		0.050 (0.038–0.062)	− 0.34	Antagonism
0.51 (0.29–0.76)		0.64 (0.36–0.95)		0.027 (0.015–0.041)	0.011 (0.0064–0.017)	2.35	Synergism
0.53 (0.31–0.79)			0.049 (0.029–0.073)	0.028 (0.016–0.042)	0.012 (0.0068–0.017)	2.23	Synergism
**Quinquenary mixtures**							
0.61 (0.39–0.87)	5.20 (3.32–7.43)	0.76 (0.48–1.08)	0.056 (0.035–0.080)	0.032 (0.021–0.046)		1.24	Synergism
0.56 (0.33–0.81)	4.78 (2.81–6.92)	0.69 (0.41–1.01)	0.052 (0.030–0.074)		0.012 (0.0072–0.018)	1.44	Synergism
0.26 (0.11–0.43)	2.21 (0.94–3.68)	0.32 (0.13–0.54)		0.014 (0.0058–0.023)	0.0057 (0.0024–0.0094)	4.26	Synergism
0.49 (0.23–0.79)	4.18 (1.96–6.75)		0.045 (0.021–0.073)	0.026 (0.012–0.042)	0.011 (0.0050–0.017)	1.79	Synergism
0.61 (0.48–0.83)		0.76 (0.59–1.04)	0.056 (0.044–0.076)	0.032 (0.025–0.043)	0.013 (0.011–0.018)	1.24	Synergism
**Senary mixture**							
0.21 (0.13–0.36)	1.79 (1.10–3.07)	0.26 (0.16–0.45)	0.019 (0.012–0.033)	0.011 (0.0068–0.019)	0.0046 (0.0029–0.0079)	4.42	Synergism

*FL* fiducial limit, *AI* additive index, *ACT* acetamiprid, *CAR* carbendazim, *AZO* azoxystrobin, *CHL* chlorpyrifos, *BIF* bifenthrin.

##### Toxic impacts of three-component mixtures

The four three-component mixtures of Cd-acetamiprid-carbendazim, Cd-acetamiprid-chlorpyrifos, Cd-carbendazim-chlorpyrifos, and Cd-chlorpyrifos-bifenthrin still displayed synergistic reactions, and their AIs ranged from 0.64 to 3.30 after 96 h of exposure. Meanwhile, the AIs for the five three-component mixtures of Cd-acetamiprid-azoxystrobin, Cd-carbendazim-azoxystrobin, Cd-carbendazim-bifenthrin, Cd-azoxystrobin-chlorpyrifos, and Cd-azoxystrobin-bifenthrin ranged from − 1.89 to − 0.33 after 96 h of exposure, suggesting antagonistic reactions. The three-component mixture of Cd-acetamiprid-bifenthrin still showed an additive reaction, and its AI was 0.18 after 96 h of exposure (Table 3).

##### Toxic impacts of four-component mixtures

The six four-component mixtures of Cd-acetamiprid-carbendazim-azoxystrobin, Cd-acetamiprid-carbendazim-chlorpyrifos, Cd-acetamiprid-carbendazim-bifenthrin, Cd-acetamiprid-chlorpyrifos-bifenthrin, Cd-carbendazim-chlorpyrifos-bifenthrin, and Cd-azoxystrobin-chlorpyrifos-bifenthrin still showed synergistic reactions, and their AIs ranged from 0.46 to 2.97 after 96 h of exposure. Meanwhile, the three four-component mixtures of Cd-acetamiprid-azoxystrobin-bifenthrin, Cd-carbendazim-azoxystrobin-chlorpyrifos, and Cd-carbendazim-azoxystrobin-bifenthrin showed an antagonistic reaction, and their AIs ranged from − 0.58 to − 0.34 after 96 h of exposure. Nevertheless, the AI of the Cd-acetamiprid-azoxystrobin-chlorpyrifos mixture was 0.41 after 96 h of exposure, implying a synergistic impact (Table 3).

##### Toxic impacts of five- and six-component mixtures

All the five-component mixtures of Cd-acetamiprid-carbendazim-azoxystrobin-chlorpyrifos, Cd-acetamiprid-carbendazim-azoxystrobin-bifenthrin, Cd-acetamiprid-carbendazim-chlorpyrifos-bifenthrin, Cd-acetamiprid-azoxystrobin-chlorpyrifos-bifenthrin, and Cd-carbendazim-azoxystrobin-chlorpyrifos-bifenthrin still displayed synergistic reactions, and their AIs ranged from 1.24 to 4.26 after 96 h of exposure. Meanwhile, the six-component mixture of Cd-acetamiprid-carbendazim-azoxystrobin-chlorpyrifos-bifenthrin still displayed a synergistic response with its AI of 4.42 after 96 h of exposure (Table 3).

### Interaction patterns of chemical combinations

**Figure 1:**
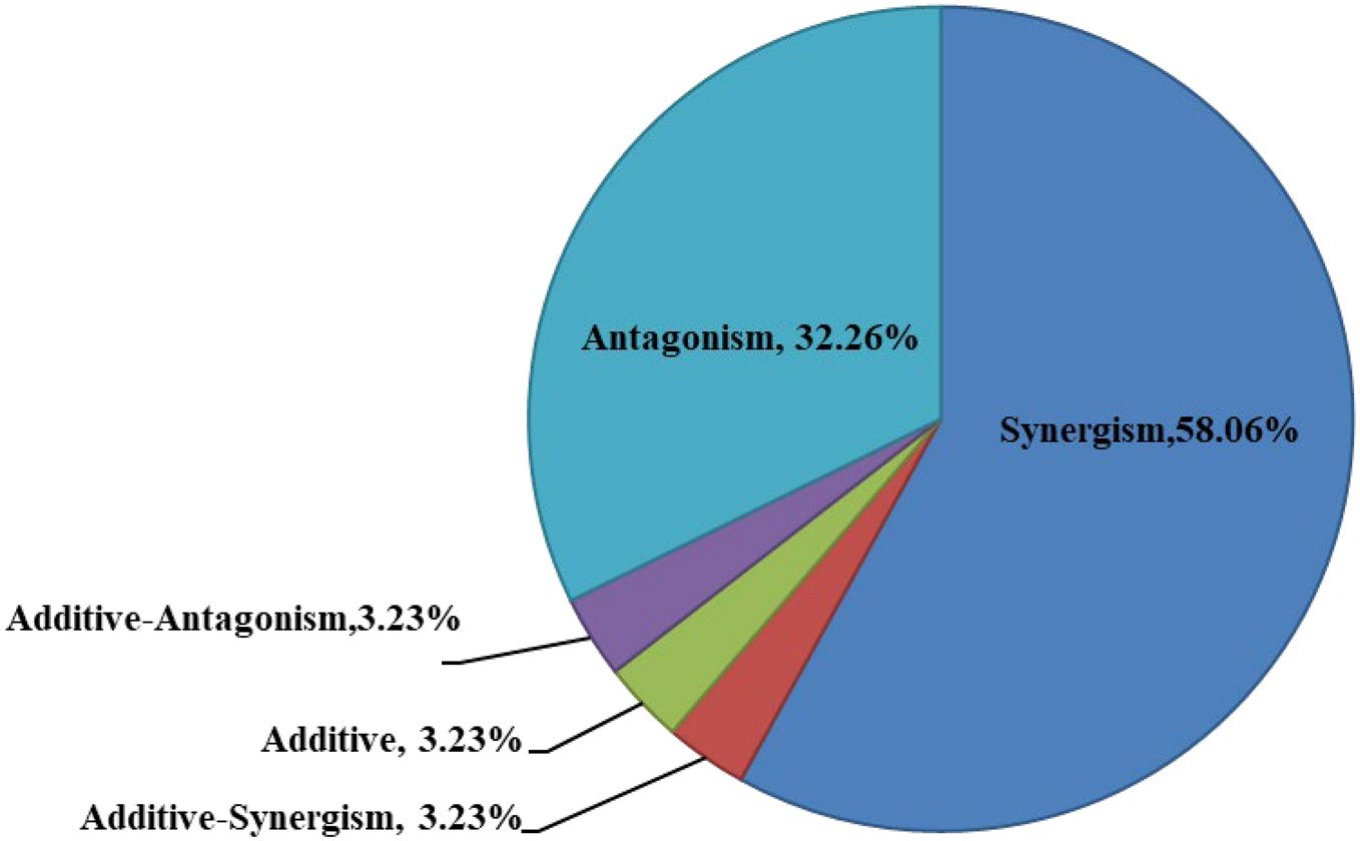
Interaction patterns of cadmium in combination with five pesticides in larval zebrafish.

In the present work, the 31 Cd-containing combinations under two different proportions were tested. Approximately 58.06% of these combinations displayed synergistic impacts. Besides, 3.23% and 3.23% of mixtures showed additive-synergistic and additive impacts on *D. rerio*, respectively. However, 3.23% and 32.26% of mixtures displayed additive-antagonistic and antagonistic reactions, respectively (Fig. 1).

## Discussion

Traditionally, the hazardous impacts of aquatic pollutants on fish are assessed by acute toxicity assay, and the main endpoint is mortality^26^. The pyrethroid insecticide bifenthrin, neonicotinoid insecticide acetamiprid, organophosphate insecticide chlorpyrifos, imidazole fungicide carbendazim, and strobilurin fungicide azoxystrobin with different modes of action have been extensively applied in agriculture worldwide^36^. Fish are mostly impaired by bifenthrin, chlorpyrifos, and azoxystrobin since organisms have a frail ability to metabolize the above-mentioned chemicals^27^. Bifenthrin can cause developmental toxicity, enantioselectivity, inflammatory cell death, and endocrine disrupting activity to *D. rerio*^13,50,51^. Previous studies have demonstrated that the 96-h LC_50_ values of Cd to the embryos and bifenthrin to the adults of *D. rerio* are 18.9 and 0.0032 mg L^−1^, respectively^28,52^, which are not similar to our findings. The discrepancy could be attributed to the difference in life stages tested^29^. Additionally, previous research has shown that the 96-h LC_50_ value of azoxystrobin and chlorpyrifos to the larvae is 0.39 and 0.41 mg L^−1^, respectively, which is similar to our data^30,31^. Acetamiprid showed the lowest acute toxicity, which might be attributed to the relatively low affinity of nicotinic acetylcholine receptors (nAChRs)^32^. To minimize unanticipated injury to the aquatic environment, more concern should be paid to bifenthrin, chlorpyrifos, and azoxystrobin in comprehensive pest management due to their frequent side effects on non-target animals.

Synergistic reactions of chemical combinations can produce severe negative impacts on the animal population, threatening the regular function of the water ecosystem^33^. To identify the possible synergistic reactions among chemicals, the toxic mechanism of chemical combinations should be clarified^34^. In the current work, the combination of chlorpyrifos and bifenthrin was included in one three-component mixture (Cd-chlorpyrifos-bifenthrin), three four-component mixtures (Cd-acetamiprid-chlorpyrifos-bifenthrin, Cd-carbendazim-chlorpyrifos-bifenthrin, and Cd-azoxystrobin-chlorpyrifos-bifenthrin), three five-component mixtures (Cd-acetamiprid-carbendazim-chlorpyrifos-bifenthrin, Cd-acetamiprid-azoxystrobin-chlorpyrifos-bifenthrin, and Cd-carbendazim-azoxystrobin-chlorpyrifos-bifenthrin), and one six-component mixture (Cd-acetamiprid-carbendazim-azoxystrobin-chlorpyrifos-bifenthrin), and all these mixtures exerted synergistic responses on *D. rerio* under two types of testing situations. Commercial formulations composed of both organophosphate insecticides and pyrethroid insecticides are available on the market, and consequent previous studies have relatively well investigated the interactive impacts of their combined pollution^35^. The ability of the organism to detoxify pyrethroid insecticide is diminished when exposed to organophosphate insecticides because of esterase inhibition, leading to synergistic toxicities of their mixture^36,37^. Thus, synergistic reactions were detected in the mixtures containing chlorpyrifos and bifenthrin in this study.

It is vital to clarify interplay patterns among agrochemicals to minimize the usage of defined combinations with adverse impacts, which is also crucial for predicting the potential toxicity of newly exploited chemicals in agriculture^38^. Acetamiprid and carbendazim are frequently manufactured as tank-mixing pesticides to have a broader spectrum of disease and pest controls and to decrease pesticide resistance^39^. Our results indicated that most of the chemical combinations containing acetamiprid and carbendazim had synergistic toxicities to *D. rerio*. Previous studies have well documented the mixture impacts of neonicotinoid insecticide and azole fungicide^40^. The azole fungicides suppress the cytochrome P450-mediated detoxification of neonicotinoid insecticides, leading to an elevation in toxicities of neonicotinoid insecticides to the organisms^41^. Thus, synergistic reactions are often detected when neonicotinoids are combined with azole fungicides^36^. To mitigate the negative impacts of acetamiprid and carbendazim on *D. rerio*, we should avoid using the combination of acetamiprid and carbendazim, and an alternative mixture should be sought to minimize the risk.

The mixture toxicity measurement exhibited that synergistic responses were also disclosed from two two-component mixtures of Cd-acetamiprid and Cd-chlorpyrifos. Cd may inhibit the activity of cytochrome P450, which is the important detoxification enzyme of agrochemicals in many organisms^42^. Such an interaction enhances the accumulation of acetamiprid and chlorpyrifos in organisms^43^. Thence, the synergistic reactions detected in the presence of Cd in combination with acetamiprid or chlorpyrifos could be attributed to that the Cd suppressed CYP450-mediated acetamiprid and chlorpyrifos detoxifications, resulting in an enhancement in their toxicity to *D. rerio*.

Besides synergistic reactions, we also found antagonistic reactions for 10 combinations of Cd-carbendazim, Cd-azoxystrobin, Cd-acetamiprid-azoxystrobin, Cd-carbendazim-azoxystrobin, Cd-carbendazim-bifenthrin, Cd-azoxystrobin-chlorpyrifos, Cd-azoxystrobin-bifenthrin, Cd-acetamiprid-azoxystrobin-bifenthrin, Cd-carbendazim-azoxystrobin-chlorpyrifos, and Cd-carbendazim-azoxystrobin-bifenthrin under two different ratios determined. Many combinations display antagonistic impacts because one of the chemicals triggers a change in toxicokinetics (such as alternative mode of action, absorption, and metabolism rates). Previous studies have reported similar findings that chemicals with a common mode of action do not necessarily act by additive toxicity as predicted^53^. In addition, the metabolism of each chemical may be motivated by other chemicals in the mixture, leading to impaired absorption into the organism^54^. Thus, we hypothesized that Cd caused the antagonistic responses in zebrafish when it was used in combination with carbendazim- or azoxystrobin-triggered detoxification of isozymes.

In the present work, we assessed the agrochemicals mixtures with equitoxic ratios and equivalent concentrations. Chemical concentrations in the water environment considerably alter, both geographically and temporally^44^. The toxicity evaluation of such multiple mixtures should incorporate a series of ratios for further determinations^20^. Chemicals may trigger direct death in the larval stages, inhibit the growth of an organism, enhance the vulnerability of fish to affect and reproduce, and negatively impair physiological or morphological courses important for excellent procreation^14,15^. Future studies should focus on whether a synergistic reaction occurs under natural conditions of these pollutants^45^. When the joint toxicity of a chemical mixture is several times greater compared with the predicted value, synergistic reactions become an elevated risk for aquatic animals^46^.

The current investigation showed that the assessment of single compounds might not reflect the real situation of these compounds in the mixture^7^. As natural water body ordinarily consists of complex chemical mixtures, toxicity data obtained from individual compounds can not accurately forecast the ecological hazard of chemicals in a practical condition^19^. Therefore, the ecosystems should be monitored by a cost-effective toxicity analysis^47^. Besides, additional studies in simulated environments using similar analytical methods are needed in each case to better reflect the ecotoxicology of chemical mixtures.

## Conclusions

Among the six detected pollutants, the highest toxicity to *D. rerio* was disclosed from bifenthrin, followed by chlorpyrifos and azoxystrobin, whereas the least toxicity was observed from acetamiprid. Moreover, 58.06% of chemical mixtures showed synergistic reactions with equivalent concentration and equitoxic ratios to *D. rerio*. Our data emphasized the complicacy of the joint toxic effect of organic and inorganic compounds in water ecosystems. Taken together, data based on individual pollutants do not reflect real field situations, which may lead to the underestimation of chemical mixtures in aquatic vertebrate populations.

## Data Availability

The datasets used or analysed during the current study are available from the corresponding author on reasonable request.
